# Intra- and Inter-Rater Reliability of Ultrasound Imaging to Measure Tensor Fasciae Latae Muscle Size

**DOI:** 10.3390/jcm14051731

**Published:** 2025-03-04

**Authors:** Elena Estébanez-de-Miguel, Luis Ceballos-Laita, Jesús Gómez-Vallejo, Sandra Jiménez-del-Barrio, Miguel Malo-Urriés

**Affiliations:** 1 Departamento de Fisiatría y Enfermería, Universidad de Zaragoza, 50009 Zaragoza, Spain; elesteba@unizar.es (E.E.-d.-M.); malom@unizar.es (M.M.-U.); 2 Departamento de Cirugía, Oftalmología, Otorrinolaringología y Fisioterapia, 42004 Soria, Spain; sandra.jimenez.barrio@uva.es; 3 Departamento de Cirugía ortopédica y Traumatología, Hospital Clínico Universitario Lozano Blesa, 50009 Zaragoza, Spain; jgomezvallejo@yahoo.es

**Keywords:** reliability, ultrasound image, tensor fascia latale

## Abstract

**Objectives**: The objectives of this study were to develop a procedure to measure the cross-sectional area and thickness of tensor fasciae latae muscle, and examine the intra- and inter-rater reliability of this procedure in healthy participants. **Methods**: The cross-sectional area and thickness of tensor fascia latae were evaluated by sonography in 14 healthy subjects (28 lower extremities) in a single session. Two examiners conducted ultrasound examinations and performed the measurements independently. Each examiner was blinded to the measurements of the other examiner. For examination, the transducer was placed transversally to the thigh at the anterior superior iliac spine level and was moved caudally along the tensor fascia latae muscle, ensuring that the volume of the muscle was in the center of the image. The examiners took ultrasound images when the maximum size was reached. **Results**: The intra-rater reliability for tensor fascia latae measurements was excellent in both examiners (cross-sectional area: ICC2,2 = 0.905–0.969, SEM = 0.29–0.82 mm^2^, MDC = 0.80–2.27; thickness: ICC2,2 = 0.965–0.985, SEM = 0.20–0.60 mm, MDC = 0.55–1.66; all *p* < 0.001). The inter-rater reliability was good for the cross-sectional area (ICC2,2 = 0.783, SEM = 0.77 mm^2^, MDC = 2.13 mm^2^). The inter-rater reliability was poor for thickness measurements (ICC2,2 = 0.445, SEM = 2.12 mm, MDC = 5.87 mm) and 2.12 mm for thickness. **Conclusions**: The procedure developed for measuring the size of the tensor fascia latae muscle with ultrasound images has demonstrated feasibility and excellent intra-rater reliability. The inter-rater reliability was good and poor for cross-sectional area and thickness measurements, respectively.

## 1. Introduction

Hip muscular atrophy is commonly associated with hip joint disorders, including acetabular impingement and osteoarthritis [1,2,3]. Furthermore, muscle atrophy has been observed to persist even after joint replacement surgery [4] or arthroscopic tenotomies [5]. Among the most affected muscles are the hip abductor and flexor muscles. Previous research has demonstrated that the volume or cross-sectional area (CSA) of key hip muscles, such as the gluteal muscles, quadriceps, iliopsoas, and tensor fasciae latae (TFL), is significantly reduced in patients with hip osteoarthritis [6,7,8]. Additionally, studies have shown that hip muscle thickness correlates more strongly with pain and functional impairment than muscle strength tests in individuals with knee osteoarthritis [9]. These findings suggest that assessing muscle size could serve as a valuable diagnostic and monitoring tool for hip joint disorders.

Magnetic resonance imaging and computed tomography are currently considered as the gold standard techniques for quantifying muscle size [10]. However, these imaging modalities are costly and may not always be readily available in clinical settings. In contrast, ultrasound imaging (USI) has emerged as a reliable, valid, noninvasive, safe, well-tolerated, portable, and accessible alternative for measuring muscle size under controlled conditions [11,12]. USI has been successfully employed to assess muscle size parameters, including CSA and thickness, in various muscle groups, such as the lumbar multifidus [13,14], abdominal muscles [15], abductor hallucis and flexor hallucis [16], and anterior tibial muscles [17].

Regarding the hip region, USI has proven to be a reliable imaging modality for measuring the CSA and thickness of anterior hip muscles, including the iliopsoas, sartorius, rectus femoris, and vastus medialis [18,19]. Additionally, it has been utilized to evaluate the gluteus medius and gluteus minimus muscles [20]. Despite the growing body of research supporting the use of USI for hip muscle assessment, there is a notable lack of evidence regarding the methodology for measuring the TFL muscle using this technique. This gap in the literature is particularly relevant given TFL’s accessibility via ultrasound and its clinical significance in hip joint disorders.

The TFL plays a crucial role in hip stability, movement, and force distribution, making its accurate assessment essential for diagnosing and monitoring musculoskeletal conditions. Moreover, as previous studies have established the association between muscle size and functional impairment, the ability to reliably measure the TFL using USI could enhance clinical decision-making and treatment planning.

Therefore, the primary objective of this study was to develop a standardized protocol for assessing the CSA and thickness of the TFL muscle using USI. Additionally, we aimed to evaluate the intra- and inter-rater reliability of this procedure to ensure its consistency and applicability in both clinical and research settings. By establishing a reliable method for measuring TFL muscle size, this study seeks to contribute to the growing body of evidence supporting USI as a valuable tool for musculoskeletal assessment, and to provide clinicians with an accessible alternative for evaluating muscle atrophy associated with hip joint disorders.

## 2. Materials and Methods

### 2.1. Participants

Fourteen healthy subjects (11 females, 3 males) were recruited from a general university population and agreed to participate in this study. Participants were excluded if they reported hip pain or a history of hip or pelvic trauma, fracture, or surgery. All enrolled subjects were included in the study, and measurements were taken for both TFL muscles (*n* = 28). The sample size exceeded the minimum required to achieve a significant intraclass correlation coefficient (ICC) value of 0.60 (1 − ß = 0.80; α = 0.05) [21].

The mean age and body mass index (BMI) of the participants were 24.35 ± 7.07 years and 22.22 ± 2.21 kg/m^2^, respectively.

This study was approved by the Ethical Committee for Clinical Research of Aragón (C.I.PI22/290, 29 June 2022). All subjects provided written informed consent, and their rights were protected throughout the study.

### 2.2. Procedure

Transverse ultrasound images of the tensor fasciae latae (TFL) muscle were obtained using two portable ultrasound imaging devices (LOGIQ e; GE Healthcare Medical System) equipped with a 10 MHz linear array transducer. Two examiners, each with more than 10 years of clinical experience in musculoskeletal USI, independently conducted the ultrasound examinations. Each examiner performed the ultrasound measurements without knowing of the other examiner’s results to ensure blinding. All ultrasonographic settings were standardized between the two examiners to maintain consistency.

Each session included four to five subjects who were examined in separate rooms to prevent any potential bias. An external assistant randomized the order of the measurements for each examiner using the Research Randomizer (Version 4.0) computer software. This process ensured that each examiner performed two measurements on both sides of each subject. The examiners followed the preassigned order indicated by the assistant and moved from room to room carrying their own ultrasound imaging device. A total of three 90 min sessions were organized to complete the examinations for all subjects.

The procedure for measuring the CSA and thickness of the TFL muscle was developed based on previous USI protocols used for assessing the hip region [22,23]. Participants were positioned in contralateral decubitus with their hips flexed at 60° and knees flexed at 90°. A cushion was placed between their legs to prevent hip internal rotation and to ensure that the femur remained in a horizontal plane. Throughout the examination, great care was taken to maintain a consistent standardized position for all subjects.

To enhance acoustic coupling and improve image clarity, a water-soluble transmission gel was applied to the ultrasound transducer. The transducer was positioned transversely over the thigh at the level of the anterior superior iliac spine and then moved caudally along the TFL muscle to ensure that the entire muscle volume was centered in the image. Notably, the size of the TFL muscle progressively increases from the anterior superior iliac spine downward. The examiners captured ultrasound images when the muscle reached its maximum size.

**Figure 1:**
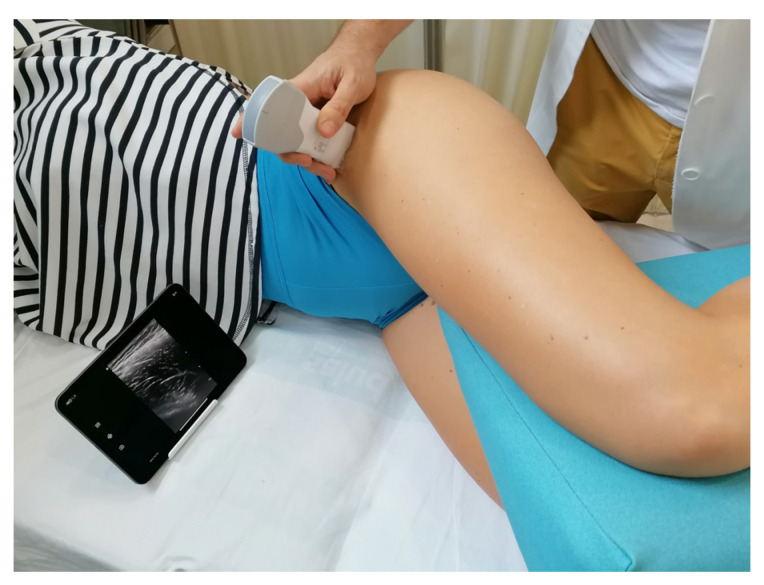
Position of the patients and transducer placement.

Figure 1 provides a representative image illustrating the standardized positioning adopted by the participants for the ultrasound measurements. This methodological approach aimed to ensure high reliability and accuracy in assessing TFL muscle morphology using USI, which may contribute to improved evaluation and monitoring of musculoskeletal conditions affecting the hip region.

### 2.3. Measurement Protocol

The measurements were performed during the ultrasound examination to ensure that the entire procedure was included in the reliability analysis. Conducting measurements in real time minimized potential variability and enhanced the consistency of the results, contributing to the accuracy and reproducibility of the study.

The CSA of the TFL muscle was determined through continuous tracing, using the hyperechoic external edge of the epimysium as the anatomical reference. This method allowed for a precise delineation of the muscle boundary, ensuring reliable measurements. Muscle thickness was assessed by measuring the anterior–posterior (AP) diameter, defined as the maximum distance between the anterior and posterior limits of the CSA tracing. This approach provided an objective and standardized evaluation of muscle morphology.

**Figure 2:**
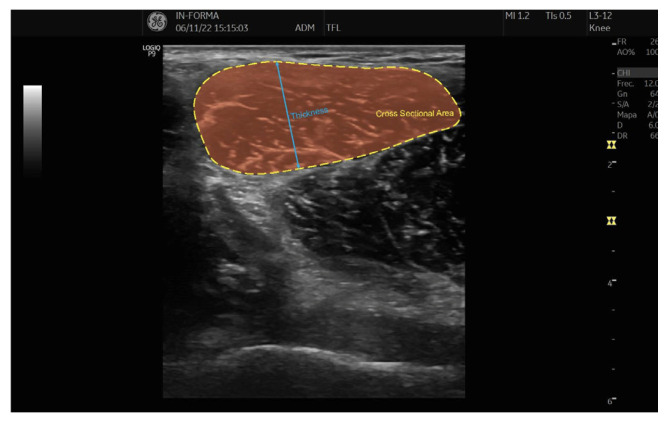
CSA and thickness of TFL.

To further illustrate these assessments, Figure 2 presents a representative ultrasound image demonstrating the CSA and thickness measurement procedures. These standardized techniques enhance the reliability of TFL muscle evaluation, offering valuable insights for both clinical and research applications related to musculoskeletal health and rehabilitation.

### 2.4. Data Analysis

Statistical analyses were carried out using SPSS version 26, developed by IBM Corporation (Armonk, NY, USA). The mean and SD values for the TFL CSA and thickness were calculated separately for two different examiners and two distinct time periods. To assess the reliability of the procedure used to measure TFL muscle size, both intra-rater and inter-rater reliability were tested using the ICCs, with a 95% confidence interval (CI) for each estimate. A two-way randomized effects model with absolute agreement (ICC2,2) was applied for the analysis. The interpretation of the ICC values followed the guidelines proposed by Portney and Watkins [24], which classify values as follows: values below 0.50 are considered poor, values between 0.50 and 0.75 indicate moderate reliability, values between 0.75 and 0.90 suggest good reliability, and values greater than 0.90 are regarded as excellent.

To assess the measurement precision, the standard error of measurement (SEM) was calculated using the formula: SEM = SD (pooled) × √(1 − ICC). Additionally, the minimum detectable change (MDC) was calculated using the formula: MDC = 1.96 × √2 × SEM, which provides an estimate of the smallest change that can be considered statistically significant.

Lastly, Bland–Altman plots were utilized to evaluate the mean difference between raters and to construct an agreement interval, providing a visual representation of the degree of agreement between measurements. A good level of agreement was defined when the mean difference between the two examiners or measurement time points was less than 5% of the overall mean value.

## 3. Results

Table 1 presents the descriptive values of the examiners’ measurements, while Table 2 displays the ICCs, 95% CIs, SEM, and MDC.

For TFL muscle measurements obtained using USI, the agreement between the two repeated measurements by each examiner was excellent, with ICC values ranging from 0.905 to 0.985. The SEM values ranged from 0.29 to 0.82 mm^2^ for CSA, and from 0.20 to 0.60 mm for thickness, indicating a low degree of measurement error. The MDC values for CSA ranged from 0.80 to 2.27 mm^2^, while for thickness, they ranged from 0.55 to 1.66 mm. These findings suggest a high level of consistency in repeated measurements from the same examiner.

In contrast, the inter-rater reliability was lower than the intra-rater reliability. The ICC for CSA measurements between examiners was 0.783, indicating good reliability, whereas the ICC for thickness measurements was 0.445, suggesting moderate reliability. The SEM for CSA was 0.77 mm^2^, and for thickness, it was 2.12 mm. The MDC values were 2.13 mm^2^ for CSA and 5.87 mm for thickness, highlighting greater variability between different examiners in assessing muscle thickness.

These results emphasize the importance of standardized protocols and examiner training to enhance the reproducibility of TFL muscle measurements using USI in both clinical and research settings.

**Figure 3:**
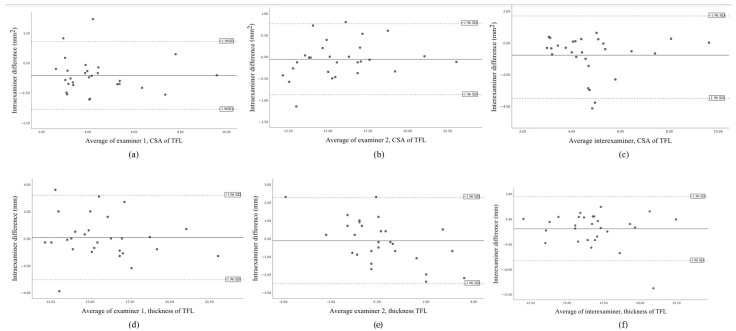
Bland–Altman plot of the intra-rater 1 agreement of the CSA of TFL (**a**), the intra-rater 2 agreement of the CSA of TFL (**b**), the inter-rater agreement of the CSA of the TFL (**c**), the intra-rater 1 agreement of the thickness of TFL (**d**), the intra-rater 2 agreement of the thickness of TFL (**e**), the inter-rater agreement of the thickness of the TFL (**f**), showing the mean difference (solid line) and 95% limits of agreement (−1.96 SD; dashed lines).

The Bland–Altman plots of the data are shown in Figure 3, illustrating an acceptable degree of agreement.

## 4. Discussion

In this study, we aimed to assess the reliability, SEM, and MDC of USI measurements of the TFL muscle. The results demonstrated excellent intra-rater reliability for all the measurements taken, indicating that the protocol used for measuring TFL muscle size was highly consistent when performed by a single examiner. However, the inter-rater reliability was found to be good for the CSA measurements of the TFL muscle, while the reliability for the thickness measurements was poor. Despite this variability in inter-rater reliability, the degree of agreement between the examiners was still considered acceptable, with the CIs indicating some uncertainty due to the smaller sample size. These findings suggest that the proposed measurement protocol is reliable when used by a single examiner, though further investigation involving larger sample sizes may be needed to confirm its broader applicability.

The results of our study showed excellent intra-rater reliability for both the CSA and thickness measurements, with narrow CIs, highlighting the precision of the method used. This indicates that the USI measurement protocol proposed in this study could serve as a reliable clinical tool for assessing the TFL muscle size, provided it is assessed by a single examiner. Our results align with those of previous studies evaluating the reliability of USI for measuring CSA in muscles such as the anterior gluteus medius, gluteus minimus, and vastus medialis [20]. In fact, the intra-rater reliability observed in our study was slightly higher than that reported by Mendis et al. [19] for CSA measurements in muscles like the iliopsoas, sartorius, and rectus femoris. However, our study could not directly compare the thickness measurements obtained by USI due to a lack of prior studies investigating intra- and inter-rater reliability and agreement for this measurement.

The measurement of muscle thickness using USI has been demonstrated to be a quick and effective screening tool for various clinical applications, such as assessing sarcopenia [25,26,27,28], evaluating function in patients with knee osteoarthritis [9], and assessing muscle activation and pain in individuals with patellofemoral pain syndrome [29]. Additionally, thickness measurements have been used to evaluate physical decline in hospitalized older adults [30]. While the thickness of the TFL muscle has not been extensively reported in the literature, we argue that this variable is important for clinical practice due to its relative ease of measurement. Furthermore, the intra-rater reliability of thickness measurements has been shown to be slightly higher than that for CSA measurements, which supports its potential use in clinical settings.

Regarding inter-rater reliability, our study found that the reliability for CSA measurements was good, while the reliability for thickness measurements was poor. Despite this variability, the degree of agreement between the raters was still acceptable in both cases. However, the CIs for inter-rater reliability were relatively wide, which may indicate a lack of precision in these measurements. This limitation could be attributed to the small sample size included in the study. Future research should involve larger, more diverse samples to better understand the variability and improve the precision of the measurements.

The fascia lata, located along the lateral iliac crest, is an important anatomical structure that has a broad attachment with contributions from the TFL muscle, the iliotibial band, and the gluteal aponeurosis. Previous studies have described USI methodologies to measure or detect pathologies associated with the fascia lata at the iliac crest [31,32], the iliotibial band syndrome [33,34], or the external snapping hip syndrome [35,36,37]. However, the specific procedure for measuring the TFL muscle size using USI has not been well established in the literature. Our study fills this gap by providing a methodology for measuring TFL muscle size, which could serve as a valuable reference for future studies.

Fatty inclusions in muscle and a reduction in muscle size reduce muscle force and quality [38]. Patients with hip OA and femoroacetabular impingement experience hip abductors muscle weakness, functional disability during dynamic weight-bearing activities, a reduced ability to activate the TFL during flexion [39,40]. The lack of existing descriptive studies on TFL muscle size measurements in healthy individuals makes our data particularly important. The results obtained in this study can serve as a reference for normal TFL muscle size and can also be used to detect true, clinically significant changes in TFL muscle size over time. The MDC values calculated in this study provide a benchmark for detecting real changes in the TFL muscle that go beyond the margin of error and variability inherent in measurement processes. As such, these values can be applied in both research and clinical practice to monitor changes in TFL muscle size, such as in rehabilitation or treatment planning for hip-related pathologies.

In this sense, the methodology designed in this study for measuring the CSA and thickness of the TFL muscle using USI has proven to be both feasible and reliable when performed by a single examiner. This technique provides a practical tool for assessing TFL muscle size and could be a valuable addition to the clinical assessment of patients with hip pathologies. Although the inter-rater reliability for thickness measurements was not as strong, the overall agreement was still acceptable, suggesting that the method may be useful for clinical applications, especially when used by a single examiner. Future research should aim to further validate these findings by including larger sample sizes and assessing the applicability of this protocol in more diverse populations.

### Limitations

Our study has several limitations. The primary limitation is the representativeness of the sample, as only healthy individuals were included in the assessment. While the key focus of this study was on reliability, and the mechanisms underlying reliability are likely similar for both patients and healthy individuals, it is important to consider that clinical application in a more diverse population should still be evaluated. Additionally, intra-rater reliability across multiple days was not assessed. Physiotherapists frequently need to monitor changes over time between treatment sessions, so further research is needed to address this aspect. Although we implemented a standardized methodology to minimize transducer pressure variability, slight variations may still occur, potentially affecting muscle thickness measurements. Future studies could consider objective pressure control mechanisms, such as pressure sensors, to further improve measurement consistency

## 5. Conclusions

This study introduces a novel methodology for accurately measuring the size of the TFL muscle using USI, a technique that has shown both feasibility and excellent intra-rater reliability. The reliability of the measurements between different raters, referred to as inter-rater reliability, was found to be good for the CSA measurements, while it was considered poor for the thickness measurements. Despite the variation in inter-rater reliability, this methodology holds significant potential for researchers who are focused on studying the morphological characteristics of the TFL muscle. The method offers a reliable and accessible approach to obtain data that could be useful for synthesizing findings across different studies, thereby advancing the scientific understanding of this muscle. Furthermore, this approach could be of great value to clinicians working in the field of musculoskeletal medicine, particularly those involved in the evaluation and treatment of patients with hip-related pathologies. By providing a practical tool for muscle assessment, it could aid in the diagnosis, monitoring, and treatment planning for patients with conditions affecting the hip and surrounding structures.

## Figures and Tables

**Table 1:** Descriptive data of the USI measurements for examiners 1 and 2.

	Examiner 1 Test	Examiner 1 Retest	Examiner 2 Test	Examiner 2 Retest
**CSA**	4.41 ± 1.66	4.32 ± 1.69	5.20 ± 1.68	5.26 ± 1.70
**Thickness**	15.81 ± 2.58	15.73 ± 2.80	17.70 ± 3.11	17.90 ± 3.44

Abbreviations: CSA: cross-sectional area.

**Table 2:** Intra- and inter-rater reliability, SEM, and MDC of the USI measurements in TFL muscle.

	Intra-Rater 1 Reliability			Intra-Rater 2 Reliability			Inter-Rater Reliability		
	ICC_3,1_ (95% CI)	SEM	MDC	ICC_3,1_ (95% CI)	SEM	MDC	ICC_3,1_ (95% CI)	SEM	MDC
**CSA**	0.969 (0.934, 0.986)	0.29	0.80	0.905 (0.794, 0.956)	0.82	2.27	0.783 (0.436, 0.908)	0.77	2.13
**Thickness**	0.985 (0.967, 0.993)	0.20	0.55	0.965 (0.924, 0.984)	0.60	1.66	0.445 (−0.093, 0.731)	2.12	5.87

Abbreviations: CSA: cross-sectional area. ICC, Intraclass Correlation Coefficient; CI, confidence interval; SEM, standard error of measurement; MDC, minimum detectable change.

## Data Availability

Data may be shared upon express request to the corresponding author.
